# Microbiological Mechanisms of Collaborative Remediation of Cadmium-Contaminated Soil with *Bacillus cereus* and Lawn Plants

**DOI:** 10.3390/plants13101303

**Published:** 2024-05-09

**Authors:** Beibei Zhou, Zehao Yang, Xiaopeng Chen, Ruonan Jia, Shaoxiong Yao, Bin Gan, Dongliang Fan, Xie Yang, Wenqian Li, Yunhan Chen

**Affiliations:** State Key Laboratory of Eco-Hydraulics in Northwest Arid Region of China, Xi’an University of Technology, Xi’an 710048, China

**Keywords:** cadmium contamination, plant–microbial combined remediation, *Bacillus cereus*, lawn plants, soil microbial ecological characteristics

## Abstract

Severe cadmium contamination poses a serious threat to food security and human health. Plant–microbial combined remediation represents a potential technique for reducing heavy metals in soil. The main objective of this study is to explore the remediation mechanism of cadmium-contaminated soil using a combined approach of lawn plants and microbes. The target bacterium *Bacillus cereus* was selected from cadmium-contaminated soil in mining areas, and two lawn plants (*Festuca arundinacea* A‘rid III’ and *Poa pratensis* M‘idnight II’) were chosen as the target plants. We investigated the remediation effect of different concentrations of bacterial solution on cadmium-contaminated soil using two lawn plants through pot experiments, as well as the impact on the soil microbial community structure. The results demonstrate that *Bacillus cereus* promotes plant growth, and the combined action of lawn plants and *Bacillus cereus* improves soil quality, enhancing the bioavailability of cadmium in the soil. At a bacterial suspension concentration of 10^5^ CFU/mL, the optimal remediation treatment was observed. The removal efficiency of cadmium in the soil under *Festuca arundinacea* and *Poa pratensis* treatments reached 33.69% and 33.33%, respectively. Additionally, the content of bioavailable cadmium in the rhizosphere soil increased by up to 13.43% and 26.54%, respectively. *Bacillus cereus* increased the bacterial diversity in the non-rhizosphere soil of both lawn plants but reduced it in the rhizosphere soil. Additionally, the relative abundance of *Actinobacteriota* and *Firmicutes*, which have potential for heavy metal remediation, increased after the application of the bacterial solution. This study demonstrates that *Bacillus cereus* can enhance the potential of lawn plants to remediate cadmium-contaminated soil and reshape the microbial communities in both rhizosphere and non-rhizosphere soils.

## 1. Introduction

Industrial production activities (such as mining, sewage irrigation, and metal smelting) have made soil heavy metal pollution one of the most important global environmental problems [1]. Heavy metal pollution has been observed to reduce the total biomass, diversity, and activity of soil microbial communities and may alter their composition [2]. The contamination of soil by cadmium has emerged as a significant and pervasive environmental issue [3]. Cadmium (Cd) is a toxic metal that threatens the human food chain [4]. Cadmium contamination not only diminishes soil productivity, leading to soil degradation, but also poses a severe threat to food security, human health, and the natural environment. Therefore, the remediation of cadmium-contaminated soil is an urgent issue [5].

Numerous technologies for the remediation of contaminated soil have been reported, such as isolation/containment technologies, solidification/stabilization technologies, and electrokinetic remediation; however, these technologies have many drawbacks, including high input, secondary pollution, and soil deterioration [2]. Additionally, most of these engineering-based technologies are not suitable for repairing large areas of contaminated soil [6]. As traditional remediation techniques like physical and chemical methods are increasingly limited in addressing soil heavy metal pollution, phytoremediation has gained significant attention as an alternative due to its ecological, economic, and sustainable advantages [7], emerging as one of the most promising remediation approaches. The key advantages of phytoremediation are lower maintenance costs, ease of application, and a public perception of this technique as a ‘green’ approach. Phytoremediation is beneficial when dealing with larger areas or when other restoration approaches might be too costly or impractical [8]. In addition, plants play a vital role in improving soil quality and optimizing the soil microbial community. Various microorganisms living in the rhizosphere can have beneficial effects on plant growth and health and increase plant biomass production [9]. Therefore, it is very important to study the changes in soil nutrients and microbial community structure during phytoremediation.

Plants are known to have different tolerances to environmental stress [10]. *Festuca arundinacea* and *Poa pratensis* are grasses belonging to the Poaceae family. They possess characteristics such as rapid growth, large biomass, wide distribution, a strong regenerative ability, and well-developed underground rhizomes and root systems. They can effectively reduce the loss and spread of pollutants [11]. In addition, *Festuca arundinacea* and *Poa pratensis* not only contribute to the remediation and beautification of the environment but also provide landscape and ecological benefits. This approach results in both esthetic and ecological benefits. The characteristic of phytoremediation is to utilize green plants to transfer/transform pollutants from the environment or render them harmless. However, the effectiveness of phytoremediation is limited due to low biomass, slow plant growth, and the low bioavailability of metals in soil [12].

Bioremediation of contaminated soil through microbial activity is a promising strategy [13]. In recent years, a large number of microorganisms with the potential to absorb Cd^2+^ have been discovered. The microorganisms detected in various studies include *Staphylococcus cohnii* L1-N1, *Bacillus cereus* CKN12 [14], *Microbacterium* sp. D2-2, and *Bacillus* sp. C9-3 [15], as well as *Enterobacter bugandensis* XY1 and *Serratia marcescens* X43 [16]. Various microbial detoxification mechanisms, including bioadsorption, bioreduction, and bioaccumulation, have been proven effective in reducing the toxicity of Cd [17]. However, similar to phytoremediation techniques, microbial remediation is also greatly influenced by external environmental factors, exhibits poor genetic stability, and demonstrates strong host specificity, leading to incomplete treatment effectiveness against various types of pollutants.

From this perspective, it is evident that individual remediation methods have limitations. Therefore, utilizing combined remediation technologies is gradually becoming a forefront hotspot. Microbially assisted phytoremediation represents a highly promising technological approach that has the potential to significantly augment the capacity of plants to uptake heavy metals [18,19]. According to a study conducted by Lanping et al. [20], *Lolium perenne* combined with heavy-metal-tolerant microorganisms can enhance the absorption of cadmium, which suggest that heavy-metal-tolerant bacteria may have the potential to reduce cadmium toxicity or enhance the tolerance of *Lolium perenne* to cadmium. Additionally, other microorganisms were proven to alter the solubility and bioavailability of soil heavy metals, thereby significantly increasing the metal concentrations in roots and shoots [21], which has great potential in the application of phytoremediation. However, the synergistic remediation of heavy-metal-contaminated soil by lawn plants and *Bacillus cereus* was not fully studied.

Therefore, in this study, we focus on the impact of microbial application on the remediation of heavy-metal-contaminated soil by lawn plants. *Festuca arundinacea* and *Poa pratensis* as green plants, alongside the cadmium-tolerant strain *Bacillus cereus,* are employed as a bacterial inoculant. The purpose of this study is (I) to evaluate the remediation effect of lawn plants combined with *Bacillus cereus* on cadmium-contaminated soil by assessing changes in soil nutrient contents and cadmium bioavailability, as well as the distribution of Cd in lawn plants; (II) to investigate the metabolic functional diversity of microbial communities in rhizosphere and non-rhizosphere soils under conditions of bacterial liquid application; and (III) to examine the relationship between plant remediation and soil microbial ecological characteristics using redundancy analysis and cluster analysis.

## 2. Results and Discussion

### 2.1. The Impact of Bacillus cereus Application Rate on Cd-Contaminated Soil

#### 2.1.1. The Effects of *Bacillus cereus* Application Rate on Soil Physicochemical Properties

**Figure 1:**
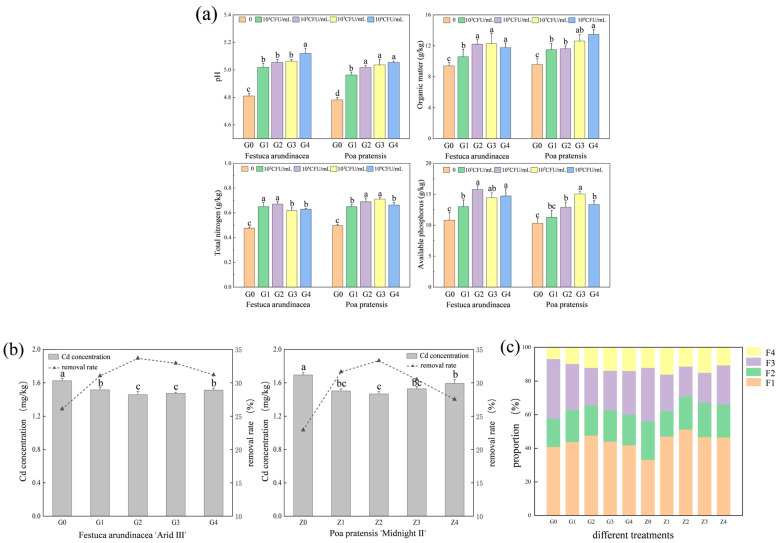
Effects of different treatments on (**a**) physical and chemical properties; (**b**) Cd concentration in soil; and (**c**) the proportion of Cd morphology in rhizosphere soil. Note: G0, G1, G2, G3, and G4 represent the concentrations of 0, 10^3^, 10^5^, 10^7^, and 10^9^ CFU/mL of *Bacillus cereus* inoculum applied to *Festuca arundinacea*, while Z0, Z1, Z2, Z3, and Z4 represent the concentrations of 0, 10^3^, 10^5^, 10^7^, and 10^9^ CFU/mL of *Bacillus cereus* inoculum applied to *Poa pratensis*. F1, F2, F3, and F4 represent different forms of soil Cd: F1 as acid-soluble, F2 as reducible, F3 as oxidizable, and F4 as residual forms. Different letters represent notable differences between the treatments at *p* < 0.05. The bars are standard deviation in the figure.

Figure 1a summarizes the changes in soil physicochemical properties under different treatments. The application of *Bacillus cereus* increased soil pH, organic matter, total nitrogen, and available phosphorus. From Figure 1a, it can be observed that compared to the control treatments G0 and Z0, the soil pH values in all treatment groups slightly increased, indicating that the application of *Bacillus cereus* in the soil could generate more alkaline substances due to its intrinsic characteristics, thereby raising the soil pH values. After planting *Festuca arundinacea* and *Poa pratensis*, the soil total nitrogen and available phosphorus contents showed a trend of increasing first and then decreasing with the increase in the bacterial liquid concentration, but they were all higher than those in the control treatment. The application of *Bacillus cereus* can increase the soil available phosphorus content, possibly due to the enhancement of soil phosphatase activity by *Bacillus cereus* by promoting the conversion of organic phosphorus to available phosphorus in the soil, which is similar to the findings of Haroun et al. [22]. Many studies have reported that soil nutrient content is closely related to plant growth. In general, high soil nutrient levels regulate the plant photosynthetic rate, stomatal conductance, and transpiration rate, promoting plant growth [23]. The inoculation of *Bacillus cereus* increased the soil available potassium, available phosphorus, and organic matter [24]. In this study, the application of *Bacillus cereus* increased the soil pH, organic matter, total nitrogen, and available phosphorus (Figure 1a). This study suggests a positive correlation between available phosphorus concentration and carbon source utilization rate [25]; rhizosphere bacteria with high metabolic activity can metabolize phosphorus-containing organic compounds in the soil, converting ineffective organic phosphorus into usable forms. Previous studies have also shown that inoculation with certain microorganisms promotes soil fertility after 60 days of greenhouse incubation [26]. The increase in soil organic matter and available phosphorus is beneficial for plant growth, especially under heavy metal stress, as nutrient-deficient environments hinder plant growth and development, limiting the progress of phytoremediation.

#### 2.1.2. The Effect of *Bacillus cereus* Application Rate on Soil Cd Content

From Figure 1b, it can be observed that different treatments are able to reduce soil Cd content to varying degrees, with overall performance indicating that *Festuca arundinacea* is less than *Poa pratensis*. In the treatment with *Festuca arundinacea*, compared to the control group G0, the soil Cd content decreased by 6.7%, 10.28%, 9.29%, and 6.94% in treatments G1, G2, G3, and G4, respectively; the soil Cd content was lowest at 1.46 mg/kg when the bacterial suspension concentration was 10^5^ CFU/mL. In the *Poa pratensis* planting treatment, compared to control Z0, treatments Z1, Z2, Z3, and Z4, respectively, reduced the soil Cd content by 11.34%, 13.49%, 9.83%, and 5.96%. The application of bacterial solution can enhance the removal efficiency of plants for the soil heavy metal Cd, and the optimal removal effect is observed at a bacterial solution concentration of 10^5^ CFU/mL; specifically, in the treatments with *Festuca arundinacea* (G2) and *Poa pratensis* (Z2), the removal rates reached 33.69% and 33.33%, respectively.

#### 2.1.3. The Effect of *Bacillus cereus* Application Rate on the Forms of Cd in Rhizosphere Soil

The bioavailability of heavy metals in soil directly affects the adsorption efficiency of plants. The heavy metal Cd in soil commonly exists in four forms: acid-soluble (F1), reducible (F2), oxidizable (F3), and residual (F4) forms. As shown in Figure 1c, the percentage content of acid-soluble cadmium is highest in the rhizosphere soil under different treatments, while the percentage content of residual cadmium is lowest. The total proportion of acid-soluble and reducible cadmium ranges from 57.76 to 70.81%, while the total proportion of oxidizable and residual cadmium ranges from 29.19 to 42.24%. With the increase in bacterial liquid concentration, the total proportion of acid-soluble and reducible cadmium in the rhizosphere soil of both types of lawn plants showed a trend of first increasing and then decreasing, and it was higher than that of the control treatment.

It is worth noting that although the total cadmium concentration in the soil decreased, the application of *Bacillus cereus* actually increased the bioavailability of cadmium. The acid-soluble and reducible forms are highly mobile and readily utilizable by plants. After inoculation with *Bacillus cereus*, the total proportion of acid-soluble cadmium and reducible cadmium in the rhizosphere soil of the two lawn plants increased by up to 13.43% and 19.82%, respectively. The increase in the percentage of bioavailable cadmium can stimulate the accumulation of cadmium in plants because the limited availability of metals in contaminated soil often hinders the efficiency of phytoremediation. In many contaminated soils, most heavy metals are bound to various organic and inorganic compounds, making them inaccessible to plants, thus becoming the primary limiting factor in the phytoremediation process [27].

### 2.2. The Effect of Bacillus cereus Application Rate on Plant Growth and Cd Absorption

**Figure 2:**
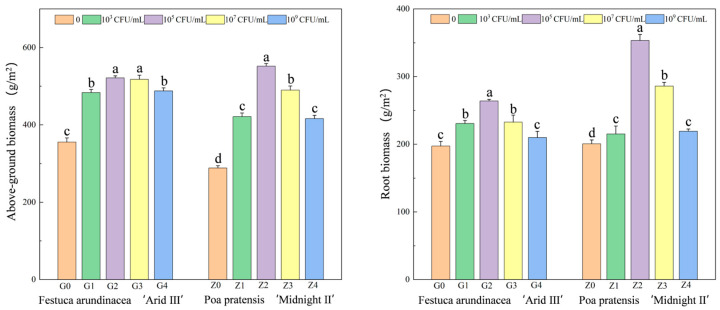
Effects of different bacterial solution concentrations on biomass coefficient of lawn plants. Note: G0, G1, G2, G3, and G4 represent the concentrations of 0, 10^3^, 10^5^, 10^7^, and 10^9^ CFU/mL of *Bacillus cereus* inoculum applied to *Festuca arundinacea*, while Z0, Z1, Z2, Z3, and Z4 represent the concentrations of 0, 10^3^, 10^5^, 10^7^, and 10^9^ CFU/mL of *Bacillus cereus* inoculum applied to *Poa pratensis*. Different letters represent notable differences between the treatments at *p* < 0.05. The bars are standard deviation in the figure.

During the 60-day growth period, both types of lawn plants grew well in the cadmium-contaminated soil, especially when the bacterial solution concentration was 10^5^ CFU/mL. As shown in Figure 2, the application of the bacterial solution significantly increased the aboveground and root biomass of both lawn plants. However, due to the differences in plant species and their tolerance to heavy metals, the changes in biomass varied among different plant parts. The aboveground and root biomass of *Festuca arundinacea* and *Poa pratensis* reached their maximum values at a bacterial solution concentration of 10^5^ CFU/mL; in the *Festuca arundinacea* treatment, the aboveground biomass increased by 46.7% compared to the control, while the root biomass increased by 33.8%. In the *Poa pratensis* treatment, the biomass increased by 81.2% and 76.1%, respectively. It can be inferred from this that *Festuca arundinacea* exhibits better tolerance to Cd compared to *Poa pratensis*.

Additionally, the Cd concentrations in both lawn plants significantly increased (Table 1), with the Cd content in the aboveground parts of *Festuca arundinacea* being up to 2.61 times higher than that in control group G0, and the Cd concentration in the roots was up to 1.41 times higher; *Poa pratensis* showed a similar trend to *Festuca arundinacea* under bacterial inoculation treatment, with the highest Cd content observed in the aboveground and root parts when the bacterial suspension concentration was 10^5^ CFU/mL, reaching 2.63 and 1.33 times that of control group Z0, respectively. The application of *Bacillus cereus* also facilitated the transport of Cd in both lawn plants and significantly increased the bioconcentration factor (BCF) of the two lawn plants. The bioconcentration factor of *Festuca arundinacea* increased to 0.607~0.906, while that of *Poa pratensis* increased to 0.423~0.519. This study demonstrates that the application of *Bacillus cereus* can enhance the transport of Cd to the aboveground parts of lawn plants and increase the storage of Cd within plant tissues, thereby achieving a reduction in soil Cd content through harvesting plant stems and leaves.

In a study conducted by Farh et al. [28], Staphylococcus and Bacillus were identified as plant growth-promoting bacteria (PGPB). These PGPB secrete growth hormones, including IAA, ACC, dehydrogenase, and iron carriers, which promote plant development [29]. After 60 days of cultivation, the application of *Bacillus cereus* indeed increased the biomass of both plant species, thereby enhancing the plants’ remediation efficiency (Table 1). The observed increase in lawn plant biomass in this study can be attributed to the secretion of IAA or the phosphate solubilization by *Bacillus cereus*. In a study conducted by Li et al. [30], it was observed that the inoculation of *Bacillus cereus* into alfalfa significantly increased plant biomass and enhanced the availability of cadmium (Cd) in soil, leading to improved efficiency of Cd removal. This study demonstrates that the application of *Bacillus cereus* can alter the bioavailability of metals and regulate metal uptake and translocation, thereby enhancing the absorption and accumulation of cadmium (Cd) in both lawn plants. The removal efficiency of heavy metals in soil is strongly influenced by the biomass of plants and the bioavailability of heavy metals. When the bioavailability of heavy metals increases, it can promote the uptake of these metals by plants, leading to higher concentrations of heavy metals in plant tissues. Therefore, this process enhances the ability of plants to extract heavy metals from the soil [31,32].

Previous studies have reported that the accumulation of Cd in plants largely depends on the bioavailable concentration of Cd. Ma et al. [33] demonstrated that inoculation with metal-resistant bacteria significantly promoted the accumulation of heavy metals in plants by improving the mobility of heavy metals in the soil. The application of *Bacillus cereus* can increase the content of acid-soluble and reducible cadmium in the soil, which may stimulate the accumulation of cadmium in both lawn plants (Figure 1c). The application of *Bacillus cereus* can activate heavy metals in rhizosphere soil, enhance the bioavailability of heavy metals in rhizosphere soil, and increase the migration ability of heavy metals, thereby enhancing the adsorption and accumulation of heavy metals by plants. Additionally, Sharma et al. [34] demonstrated that Bacillus spp. enhance the bioavailability of heavy metals by producing organic acids and polysaccharides. Previous studies have shown that the accumulation and uptake of heavy metals (HMs) by plants highly depend on their bioavailability in the soil [35]. Due to the increased bioavailability of HMs, the phytoremediation efficiency of sunflowers for uranium (U)- and cadmium (Cd)-contaminated soil significantly improved in pot experiments [36].

### 2.3. The Effect of Bacillus cereus Application Rate on the Microbial Structure of Heavy-Metal-Contaminated Soil

#### 2.3.1. The Variation in Bacterial Community Alpha Diversity between Rhizosphere and Non-Rhizosphere Soil

Heavy metal pollution can continue to influence soil physicochemical properties, thereby affecting the community structure and functionality of soil microorganisms [37,38]. By calculating the Alpha diversity indices of OTU sequences in Cd-contaminated soil under different treatments, we analyzed the changes in microbial diversity and richness in both rhizosphere and non-rhizosphere soil induced by different bacterial liquid concentrations. As shown in Table 2, in non-rhizosphere soil of both lawn plants, the Chao1 index, Shannon index, and Simpson index increased overall under each bacterial treatment, indicating an improvement in the metabolic diversity of the treated samples. This might be attributed to the availability of soil nutrients and other soil environmental conditions, including higher nutrient content and enzyme activity in the soil [39]. In rhizosphere soil, under bacterial treatment for both lawn plants, there was no significant change in the Simpson index, but the Chao1 index and Shannon index decreased compared to the control treatment, indicating that the application of *Bacillus cereus* might reduce the richness of bacterial communities in rhizosphere soil. It is speculated that *Bacillus cereus* can rapidly adapt to the rhizosphere environment of plants, becoming a dominant microbial community, and inhibiting the growth and reproduction of most other microorganisms.

#### 2.3.2. The Changes in the Relative Abundance of Bacterial Species

**Figure 3:**
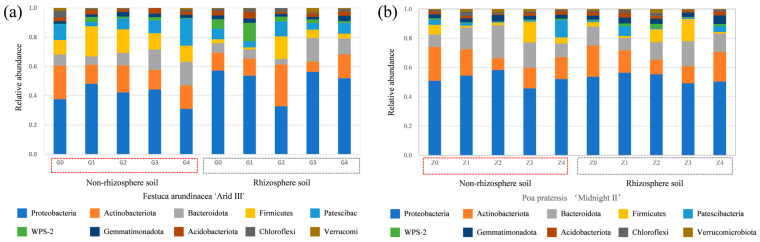
Relative abundance of the top 10 dominant microorganisms of bacteria at the phylum level in rhizosphere soil and non-rhizosphere soils under different treatments (%): (**a**) *Festuca arundinacea;* (**b**) *Poa pratensis*. Note: G0, G1, G2, G3, and G4 represent the concentrations of 0, 10^3^, 10^5^, 10^7^, and 10^9^ CFU/mL of *Bacillus cereus* inoculum applied to *Festuca arundinacea*, while Z0, Z1, Z2, Z3, and Z4 represent the concentrations of 0, 10^3^, 10^5^, 10^7^, and 10^9^ CFU/mL of *Bacillus cereus* inoculum applied to *Poa pratensis*.

The relative abundance results of the top ten bacterial species at the phylum level across all samples indicate significant changes in the composition of microbial communities in both rhizospheric and non-rhizospheric soils following *Bacillus cereus* inoculation. The phyla *Proteobacteria*, *Actinobacteriota*, *Bacteroidota*, *Firmicutes*, and *Patescibacteria* are the most abundant across all samples. In the non-rhizospheric soil of *Festuca arundinacea* (Figure 3a), they collectively account for 90.27~95.48% of the total abundance. In the rhizospheric soil, they collectively account for 77.32~90.78% of the total abundance. In the non-rhizospheric soil of *Poa pratensis* (Figure 3b), their total abundance collectively ranges from 80.5 to 90.7%, while in the rhizospheric soil, their total abundance collectively ranges from 81.5 to 93.2%. However, the distribution of each phylum varies depending on different soil locations and treatments. The phylum *Proteobacteria* tends to be more enriched in the rhizosphere across all treatments. *Proteobacteria*, which are Gram-negative bacteria, are abundant in harsh environments. They are characterized by an outer layer of lipopolysaccharides in their cell wall, which helps to sequester heavy metals from the external environment, thereby reducing the harmful toxicity of heavy metals and conferring a high degree of tolerance to them [40,41,42]. Under different treatments, the changes in various phyla differ. As shown in Figure 3a, compared to control G0, the relative abundance of *Actinobacteriota* decreased in both rhizosphere and non-rhizosphere soils of *Festuca arundinacea* after the application of the bacterial solution. Conversely, the relative total abundance of *Proteobacteria*, *Firmicutes*, and *Bacteroidota* increased. With the increasing bacterial solution concentration, the relative total abundance showed a trend of initially increasing and then decreasing. As depicted in Figure 3b, the relative abundance of *Actinobacteriota* decreased in both rhizosphere and non-rhizosphere soils of *Poa pratensis* after the application of the bacterial solution, similar to *Festuca arundinacea*. This may be attributed to the decrease in the soil cadmium concentration after the remediation treatment, as the abundance of *Actinobacteriota* in the soil is positively correlated with the concentration of heavy metals [43]. *Actinobacteria*, as rhizospheric bacteria, have been widely studied. They are capable of decomposing various organic compounds and utilizing different carbohydrates as energy sources [44].

**Figure 4:**
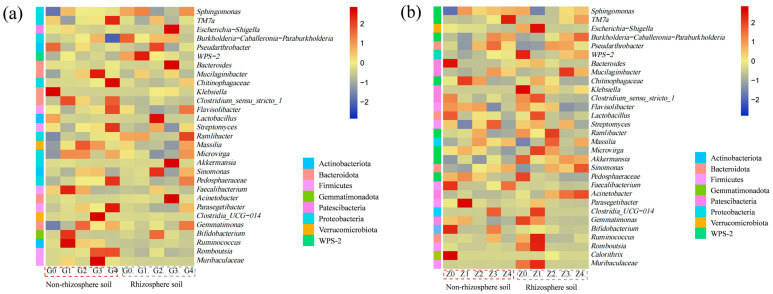
Heatmap of the top 30 genera of rhizosphere and non-rhizosphere soil bacteria under different treatments: (**a**) *Festuca arundinacea;* (**b**) *Poa pratensis*. Abundance is expressed as color intensity, reflecting the proportion of the total number of valid sequences in each group. Vertical: treatment. Horizontal: annotation information for species. Note: G0, G1, G2, G3, and G4 represent the concentrations of 0, 10^3^, 10^5^, 10^7^, and 10^9^ CFU/mL of *Bacillus cereus* inoculum applied to *Festuca arundinacea,* while Z0, Z1, Z2, Z3, and Z4 represent the concentrations of 0, 10^3^, 10^5^, 10^7^, and 10^9^ CFU/mL of *Bacillus cereus* inoculum applied to *Poa pratensis*.

In order to further understand the correlation within species, a hierarchical clustering heatmap analysis was conducted to illustrate the microbial community profile at the genus level (Figure 4). The heatmap displays an overview of the 30 most abundant genera, illustrating the impact of the inoculation on the composition of both rhizospheric and non-rhizospheric bacterial communities. Based on the relative abundance results of bacterial phyla, most bacteria in each treatment belong to *Proteobacteria* and *Firmicutes*; the composition of bacterial communities changed significantly with the application of bacterial liquid treatments. In the non-rhizospheric area of *Festuca arundinacea* (Figure 4a), compared to the G0 treatment, the relative abundance of *Pseudarthrobacter*, *Klebiella*, and *Lactobacillus* decreased after the application of bacterial liquid. Conversely, the relative abundance of genera such as *Sphingomonas*, *Mucilaginibacter*, *Ramlibacter*, *Massilia*, *Gemmatimonas*, and *Microvirga* increased. Research has found that *Sphingomonas* can promote the secretion of plant hormones such as indole-3-acetic acid, participate in nitrogen cycling, and be applied to the degradation of pollutants and the remediation of heavy-metal-contaminated soils [45,46]. In the rhizospheric area of *Festuca arundinacea*, following the application of the bacterial solution, there was an increase in the relative abundance of genera such as *Lactobacillus* (which produces lactic acid as a byproduct of glucose metabolism), *Pseudarthrobacter* (known for pollutant degradation), *Sinomonas*, and *Bifidobacterium* (beneficial bacteria capable of synthesizing various vitamins).

Additionally, the distribution of the microbial community structure responded differently to the simultaneous application of different concentrations of bacterial solution. In the non-rhizospheric area of *Poa pratensis* (Figure 4b), after the application of the bacterial solution, the relative abundance of dominant genera such as *Bacteroides*, *Faecalibacterium*, *Bifidobacterium*, and *Calorithrix* decreased in the Z0 treatment. The distribution of the microbial community structure in the Z1 treatment mainly focused on *Chitinophagaceae* and *Parasegetibacter* (Cd-tolerant, promoting plant growth), while in the Z2 treatment, it mainly focused on *Ramlibacter* and *Massilia*. In the Z3 treatment, it mainly concentrated on *Pseudarthrobacter*, WPS-2, *Mucilaginibacter* (Cd-resistant strains), *Clostridia*_UCG-014, and *Ruminococcus*. In the rhizospheric area of *Poa pratensis*, after the Z1 treatment, the relative abundance of *Microvirga* and *Lactobacillus* increased compared to the Z0 control. Following the Z2 treatment, *Massilia* (with a phosphorus-solubilizing ability, metal-tolerant) and *Ramlibacter* (beneficial bacteria) showed increased abundance compared to the control. After the Z3 and Z4 treatments, the abundance of *Acinetobacter*, *Pseudarthrobacter* (pollutant-degrading bacteria), *Sinomonas*, and *Mucilaginibacter* (metal-tolerant strains, involved in the remediation of heavy metal pollution) increased compared to the control. These research findings indicate that the application of *Bacillus cereus* leads to increased diversity among bacterial populations. This diversity promotes the stability of microbial communities, thereby aiding in alleviating the toxicity of heavy-metal-contaminated soils.

#### 2.3.3. Biomarker Identification and LEfSe Analysis

**Figure 5:**
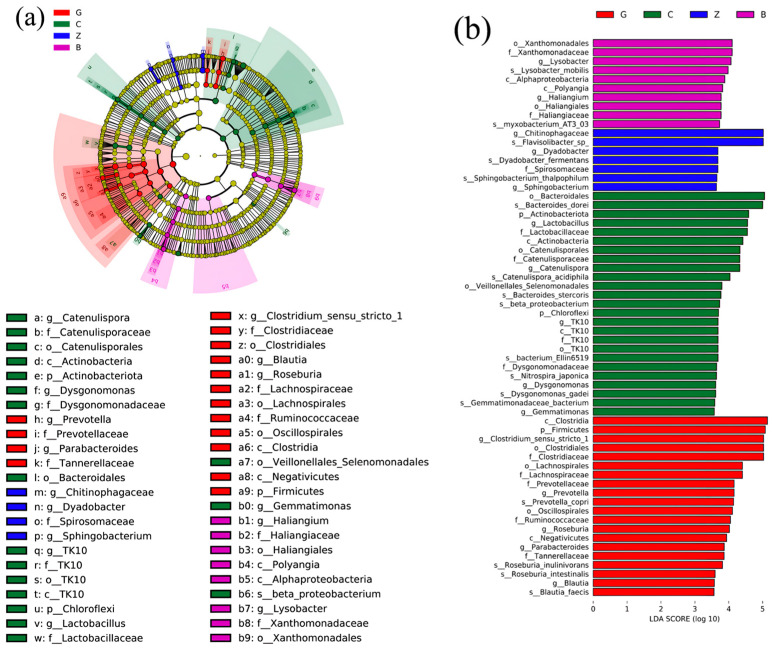
LEFse analysis of bacterial community of each group: (**a**) taxonomic cladistics of bacteria community; (**b**) LDA distribution of bacterial community. Note: G and C groups represent non-rhizospheric and rhizospheric soils of *Festuca arundinacea*; Z and B groups represent non-rhizospheric and rhizospheric soils of *Poa pratensis*. In the phylogenetic tree, nodes from inner to outer represent taxonomic categories of bacterial communities from phylum to genus, where p, c, o, f, g, and s represent phylum, class, order, family, genus, and species levels, respectively. The size of the nodes is proportional to their relative abundance. The length of the bars in the LDA value histogram reflects the contribution of species abundance to the differential effect size.

Using the LEfSe 1.1.01 software, bacterial biomarkers at the phylum to genus level were identified for different treatments, including the cladogram (Figure 5a) and the LDA distribution histogram (Figure 5b). The results indicate significant differences in the enriched species communities between the rhizospheric and non-rhizospheric zones of *Festuca arundinacea* and *Poa pratensis.* Taxa such as *Firmicutes*, *Clostridia*, *Negativicutes*, *Clostridiales*, *Lachnospiraceae*, *Prevotellaceae*, *Oscillospirales*, *Clostridiaceae*, *Lachnospiraceae*, *Tannerellaceae*, *Prevotella*, *Parabacteroides*, *Roseburia*, *Blautia*, and *Clostridium_sensu_stricto*_1 were specifically enriched in the non-rhizospheric soil of *Festuca arundinacea. Actinobacteriota* and *Chloroflexi* phyla, as well as the *Actinobacteria* class, the TK10 order, the *Catenulisporales* order, the TK10 family, the *Dysgonomonadaceae* family, the *Catenulisporaceae* family, the *Lactobacillaceae* family, the *Catenulispora* genus, the *Dysgonomonas* genus, the *Lactobacillus* genus, and the *Gemmatimonas* genus are specifically enriched in the rhizosphere soil of *Festuca arundinacea*. The family *Spirosomaceae* and the genera *Chitinophagaceae*, *Dyadobacter*, and *Sphingobacterium* were specifically enriched in the non-rhizospheric soil of *Poa pratensis*. The genera *Polyangia* and *Lysobacter*, along with the class *Alphaproteobacteria*, the order *Haliangiales*, the family *Haliangiaceae*, and the family *Xanthomonadaceae*, were specifically enriched in the rhizospheric soil of *Poa pratensis*. These results indicate that different treatments lead to differences in the indigenous microbial taxa and their activity expression between rhizospheric and non-rhizospheric soils.

### 2.4. The Response Relationship between Plant Remediation and Soil Microbial Ecological Characteristics

**Figure 6:**
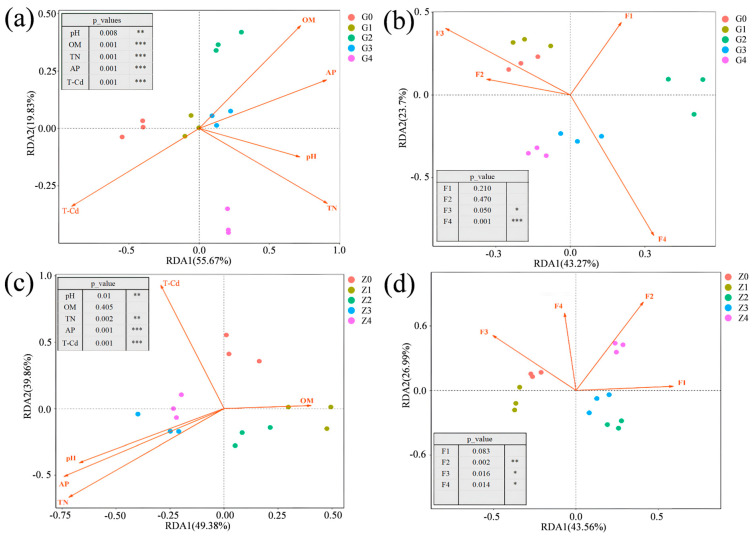
RDA of environmental factor counts in rhizosphere soils for bacteria: (**a**,**b**) Soil properties and soil bacterial communities of tall *Festuca arundinacea*. (**c**,**d**) Soil properties and soil bacterial communities of *Poa pratensis*. F1, F2, F3, and F4 represent different forms of soil Cd: F1 as acid-soluble, F2 as reducible, F3 as oxidizable, and F4 as residual forms. *p* ≤ 0.001 is represented by three symbols (***), 0.001 < *p* ≤ 0.01 is demonstrated by two asterisks (**), and 0.01 < *p* ≤ 0.05 is demonstrated by a single asterisk (*).

The relationship between plant remediation and plant microecological characteristics is closely associated with soil physicochemical properties, heavy metal content, and bacterial community structure. To fully evaluate the relative impacts of soil physicochemical parameters (pH, OM, TN, AP) and heavy metal Cd concentration, as well as heavy metal forms (F1, F2, F3, F4), on the abundance and diversity of microbial communities in heavy-metal-contaminated farmland, redundancy analysis (RDA) was conducted (Figure 6). The results indicate that pH, OM, TN, AP, and Cd, as well as Cd forms F3 and F4, are the main factors influencing the bacterial community structure in *Festuca arundinacea* (*p* < 0.05) (Figure 6a,b). The main factors influencing the bacterial community structure in *Poa pratensis* are pH, TN, AP, and Cd, as well as Cd forms F2, F3, and F4 (*p* < 0.05) (Figure 6c,d). The results indicate that the soil microbial community structure is driven by soil nutrients such as TN and AP [47].

**Figure 7:**
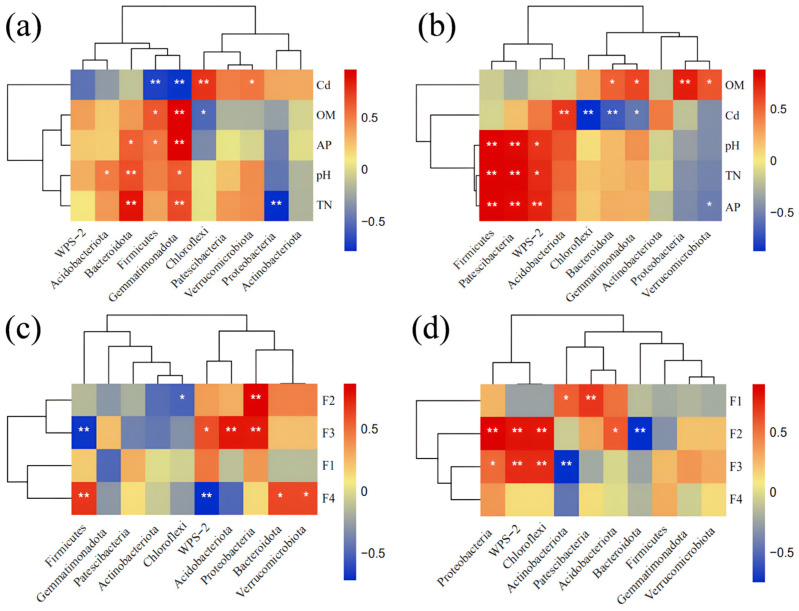
The relationship between soil physicochemical properties and microbial community composition at the phylum level for (**a**) non-rhizospheric soil of *Festuca arundinacea*, (**b**) non-rhizospheric soil of *Poa pratensis*, (**c**) rhizospheric soil of *Festuca arundinacea*, and (**d**) rhizospheric soil of *Poa pratensis*. *p* ≤ 0.01 is demonstrated by two asterisks (**), and 0.01 < *p* ≤ 0.05 is demonstrated by a single asterisk (*).

By combining the Spearman correlation heatmap with UPGMA clustering analysis, we investigated the relationship between soil properties and soil microbial community composition at the phylum level in non-rhizosphere and rhizosphere soils (Figure 7). In *Festuca arundinacea* soil, TN, pH, and AP were significantly positively correlated with *Bacteroidota* (*p* < 0.05), while soil AP and OM were significantly positively correlated with *Firmicutes*. Soil Cd content showed a significant negative correlation with *Firmicutes* and *Gemmatimonadota* (Figure 7a). In *Poa pratensis* soil, TN, pH, and AP were identified as key environmental factors positively influencing *Firmicutes* and *Patescibacteria* (*p* < 0.05). Soil T-Cd content showed a negative impact on *Bacteroidota*, exhibiting a significant negative correlation (Figure 7b). In the rhizospheric soil of *Festuca arundinacea*, oxidizable Cd content (F2) and reducible Cd content (F3) were significantly positively correlated with *Proteobacteria*. Residual Cd content (F4) showed a significant positive correlation with *Firmicutes* and *Bacteroidota* (Figure 7c). In the rhizospheric soil of *Poa pratensis*, the oxidizable Cd content (F2) showed a significantly positive correlation with *Proteobacteria* (*p* < 0.01) and a significant negative correlation with *Bacteroidota* (Figure 7d).

The interaction between roots and microbes plays a significant role in the phytoremediation of heavy-metal-contaminated soil. Microbes are pivotal players in the bioremediation of polluted soil, with certain functional microbes capable of reducing soil contamination levels [48]. Several growth parameters of the plant *H. Pennisetum*, including shoot length, biomass, and dry weight, indicate that inoculation with *Bacillus* significantly improves plant growth in the soil [49]. Compared to the CK treatment, the application of *Bacillus cereus* increased the diversity of bacterial communities in the non-rhizospheric soil of the lawn plants but reduced the richness of bacterial communities in the rhizospheric soil (Table 2). Meanwhile, the application of *Bacillus cereus* significantly increased the relative abundance of the phyla *Proteobacteria* and *Firmicutes* in the soil (Figure 3). *Proteobacteria* and *Firmicutes* have also been found to be dominant phyla in mine soil due to their possession of heavy-metal-tolerant gene clusters [50]; the ability of these phyla to coexist in extreme environments is believed to have the potential for remediating heavy metal pollution. In conclusion, *Bacillus cereus* significantly increased the relative abundance of dominant genera associated with improving soil nutrients, reducing soil Cd concentration, and altering the form of heavy metals. These factors may partly explain how the application of *Bacillus cereus* promotes the growth of two types of lawn plants and enhances their uptake of Cd, thus contributing to the increased efficiency of microbial-enhanced phytoremediation of Cd-contaminated soil.

## 3. Materials and Methods

### 3.1. Experimental Materials

The soil used in this experiment was collected from the 0–20 cm layer of the heavily metal-contaminated area in Dafanshan Mining Area, Lujiang County, Anhui Province. The soil texture is silty clayey loam (13.25% clay, 52.84% silt, and 22.48% sand). The soil was passed through a 2 mm sieve and then placed in sealed bags for storage. The basic physicochemical properties of the initial soil samples at the sampling points are shown in Table 3. The strains were screened from the soil samples of this experiment and identified as *Bacillus cereus* [51]. Our experiment shows that this strain can grow well in a medium with a cadmium concentration of 50 mg/L and exhibits excellent tolerance to high cadmium concentrations.

In the pot experiment, two common lawn plants were selected as the test subjects: *Festuca arundinacea* ‘Arid Ⅲ’ and *Poa pratensis* ‘Midnight Ⅱ’.

### 3.2. Experimental Design and Sample Collection

The pot experiment was conducted in the greenhouse of Xi’an University of Technology, located in Xi’an City, Shaanxi Province, China. The tested bacterial strains were inoculated into beef extract peptone broth. The inoculated broth was then incubated at 30 °C and 150 r/min for 72 h. After incubation, it was centrifuged at 5000 r/min for 10 min, and the supernatant was discarded, preparing suspensions of the test strains at different concentrations. The bacterial pellets were resuspended in 20 mL of sterile water. The duration of the pot experiment was 60 days after seeding, with temperature settings of 25 °C during the day (12 h) and 15 °C at night (12 h) in the growth chamber. There were 10 treatments in total, with each treatment replicated 3 times, resulting in a total of 30 pots. In total, 5.5 kg of contaminated soil from the mining area was weighed and placed into polyethylene plastic pots (43 cm long, 19 cm wide, and 14 cm high, with four drainage holes at the bottom). Each pot was then fertilized with 1.21 g of potassium dihydrogen phosphate and 1.28 g of urea as basal fertilizer. Additionally, 0.5 g of lawn plant seeds were sown and covered with a thin layer of soil on the surface. Throughout the entire growth period, water was periodically supplemented using the weighing method to maintain soil moisture at 60~70% of field capacity. Additionally, the positions of the pots were regularly changed to eliminate the effects of light exposure on the growth of the lawn plants. After the seeds germinated, different concentrations of the tested bacterial suspension were inoculated into the root zone of the plants, with the concentrations specified in Table 4.

After 60 days of growth, soil and plant samples were collected from the lawn plants. We cleaned the aboveground parts and root systems of the plants, then weighed them separately. The plant samples were then oven-dried at 105 °C for 30 min, followed by drying at 80 °C until a constant weight was achieved. The dried plant samples were pulverized, ground, and stored in bags. Soil samples were divided into rhizosphere soil and non-rhizosphere soil. Whilst separating the roots, the rhizosphere soil was collected in a plastic bag by shaking the roots vigorously. The soil outside the root zone was collected as the non-rhizosphere soil [52]. A portion of the soil samples was air-dried and passed through a 1 mm nylon sieve for the determination of soil organic matter, available phosphorus, and other nutrient contents. Another portion of the soil samples was passed through a 0.15 mm nylon sieve for the determination of heavy metal content in the soil. Another portion of the soil samples underwent freeze-drying at low temperature (Beijing Songyuan, Beijing, China); the soil samples were ground using a fully automatic cryogenic grinder (JXFSTPRP-CL, Shanghai Jingxin, Shanghai, China), sieved, and stored in a −80 °C freezer for subsequent high-throughput sequencing analysis of soil microbial communities.

### 3.3. Measurement Methods

#### 3.3.1. Soil Physicochemical Properties

The pH of the collected soil samples was measured using a pH meter (Mettler Toledo, Switzerland); the suspension had a soil/water ratio of 1:2.5 (mass/volume) [53]. Soil organic matter (OM) was determined using the potassium dichromate volumetric method with external heating. Total nitrogen (TN) is determined using the Kjeldahl method, available phosphorus (AP) in soil is measured with a SmartChem fully automated discrete chemistry analyzer (Smartchem 450, AMS Alliance, Rome, Italy), and the total Cadmium (T-Cd) content (C_1_)in soil is determined by the digestion method using hydrochloric acid, nitric acid, hydrofluoric acid, and perchloric acid., while the modified BCR sequential extraction method was employed to determine the content of different forms of cadmium (C_2_), which were then measured using graphite furnace atomic absorption spectrometry. The calculation formula for the relative content of each form of Cd (C_3_) is as follows:

C_3_ = C_2_ × 100%/C_1_ (1)

#### 3.3.2. Plant Biomass and Heavy Metal Accumulation

The plant samples were oven-dried at 105 °C for 30 min to terminate biological activity, followed by drying at 80 °C to a constant weight, and then the plant biomass was determined. The determination of heavy metal content was divided into two parts: aboveground parts and roots. In total, 0.200 g (accurate to 0.0001 g) of dried plant samples was weighed and transferred into digestion tubes. After soaking overnight in 10 mL of HNO_3_ and HClO_4_ (7:1, *v*/*v*) solution, the digestion tubes were placed in a graphite digestion instrument (EH-2542, Beijing Auwii Instrument Co., Ltd., Beijing, China) and an automatic heating program was set as follows: digestion was carried out at 120 °C for 60 min, followed by digestion at 160 °C for 30 min and, finally, at 180 °C for 15 min. The Cd content in the plant samples was determined using graphite furnace atomic absorption spectrometry, with each treatment replicated three times. The bioconcentration factor (BCF) and translocation factor (TF) of heavy metal Cd in plants were calculated using the following formulas:

BCF = C_t_/C_s_ (2)

TF = C_aboveground_/C_root_ (3)

where C_t_ and C_s_ are the Cd concentration in plant tissues and soils; C_aboveground_ and C_root_ are the Cd concentration in the plants’ aboveground part and root part.

#### 3.3.3. Soil Microbial Structure

DNA extraction was performed on soil samples from metal-contaminated layers (fresh samples) and soil samples after different remediation treatments. The bacterial 16S rRNA gene amplicon sequences were determined through sequencing. Total DNA was extracted from the filter membranes using the FastDNA Spin Kit (MP Biomedicals, Santa Ana, CA, USA), following the instructions provided in the manual. The quality and concentration of the extracted DNA were measured using the NanoDrop ND-1000 ultraviolet–visible spectrophotometer (Thermo Fisher Scientific, Waltham, MA, USA). The primers 515F (5′-CCTAYGGGRBGCASCAG-3′) and 806R (5′-GGACTACNNGGG TATCTAAT-3′) were used to amplify the V3-V4 region of the 16S rRNA gene via PCR. The PCR products were sent to Novogene Co., Ltd. (Tianjin, China) for subsequent library construction and sequencing on the Illumina MiSeq high-throughput sequencing platform provided by the company (Illumina, Kapa Biosciences, Woburn, MA, USA). The remaining sequences after filtering chimeras using Usearch 11 software were clustered into operational taxonomic units (OTUs) based on a 97% similarity threshold. Subsequently, data from each sample were normalized, and the Alpha diversity indices such as Chao1, Shannon, and Simpson were calculated using Qiime software (Version 1.9.1).

### 3.4. Statistical Analysis

All experimental data were statistically processed using Excel 2016 software; the data were subjected to a one-way analysis of variance (ANOVA) using the SPSS 26.0 software (SPSS, Inc., Chicago, IL, USA) at a significance level of *p* ≤ 0.05. Graphs were generated using Origin 2021 software. The correlation between microbial communities and soil environmental factors was analyzed using R 4.2.2. The Canoco 4.5 software was employed for redundancy analysis (RDA) to determine the important environmental factors influencing the microbial community structure.

## 4. Conclusions

*Bacillus cereus* inoculation promotes the growth and Cd accumulation of lawn plants (*Festuca arundinacea* A‘rid III’ and *Poa pratensis* M‘idnight II’). This study demonstrated that the application of *Bacillus cereus* reduced the Cd content in soil to some extent and improved the soil quality. Additionally, the application of *Bacillus cereus* promoted the transformation of Cd in rhizospheric soil from the oxidizable and residual states to the acid-soluble and reducible states. In the *Festuca arundinacea* and *Poa pratensis* treatments, the bioavailable Cd content in rhizospheric soil increased by up to 13.43% and 26.54%, respectively. Moreover, the application of *Bacillus cereus* enhanced the absorption of cadmium by plants. Both lawn plants demonstrated the ability to absorb significant amounts of cadmium from the soil and translocate it into their tissues after the application of *Bacillus cereus*. The optimal remediation treatment was observed when the bacterial liquid concentration was 10^5^ CFU/mL. The bioconcentration factor (BCF) of *Festuca arundinacea* and *Poa pratensis* increased by 0.906 and 0.519 times, respectively, and the translocation factor (TF) of cadmium in plant tissues reached its maximum values of 0.467 and 0.339, respectively. Additionally, *Bacillus cereus* increased the diversity of bacterial communities in non-rhizospheric soil, while reducing diversity in rhizospheric soil. The application of *Bacillus cereus* treatment reduced the proportion of *Actinobacteriota* in both the rhizospheric and non-rhizospheric soil of lawn plants, while enhancing the relative abundance of ecologically beneficial microorganisms in the soil. This study confirms the significant potential and prospects of using lawn plants in conjunction with *Bacillus cereus* for the remediation of cadmium-contaminated soil.

## Figures and Tables

**Table 1:** Effects of different concentrations of bacterial solution on Cd concentrations in lawn plants.

Lawn Plants	Handle	Concentration (CFU/mL)	Cd Concentration (mg/kg)		Translocation Factor (TF)	Bioconcentration Factor (BCF)
			Aboveground	Root		
*Festuca arundinacea* ‘Arid Ⅲ’	G0	0	0.481 ± 0.012 ^d^	1.907 ± 0.061 ^d^	0.255 ± 0.003 ^d^	0.431 ± 0.006 ^c^
	G1	10^3^	0.753 ± 0.013 ^c^	2.557 ± 0.08 ^b^	0.294 ± 0.006 ^c^	0.603 ± 0.009 ^b^
	G2	10^5^	1.256 ± 0.139 ^a^	2.682 ± 0.093 ^a^	0.467 ± 0.039 ^a^	0.898 ± 0.031 ^a^
	G3	10^7^	0.992 ± 0.018 ^b^	2.489 ± 0.039 ^c^	0.398 ± 0.007 ^b^	0.877 ± 0.010 ^a^
	G4	10^9^	0.756 ± 0.014 ^c^	2.557 ± 0.082 ^b^	0.308 ± 0.005 ^c^	0.673 ± 0.011 ^b^
*Poa pratensis* ‘Midnight Ⅱ’	Z0	0	0.323 ± 0.027 ^e^	1.895 ± 0.031 ^d^	0.170 ± 0.012 ^d^	0.221 ± 0.006 ^d^
	Z1	10^3^	0.651 ± 0.038 ^b^	2.385 ± 0.069 ^b^	0.273 ± 0.014 ^b^	0.530 ± 0.013 ^b^
	Z2	10^5^	0.851 ± 0.043 ^a^	2.526 ± 0.136 ^a^	0.339 ± 0.031 ^a^	0.499 ± 0.029 ^a^
	Z3	10^7^	0.529 ± 0.018 ^c^	2.065 ± 0.045 ^c^	0.256 ± 0.014 ^c^	0.452 ± 0.015 ^c^
	Z4	10^9^	0.469 ± 0.041 ^d^	1.825 ± 0.026 ^d^	0.257 ± 0.021 ^c^	0.456 ± 0.017 ^c^

Note: G0, G1, G2, G3, and G4 represent the concentrations of 0, 10^3^, 10^5^, 10^7^, and 10^9^ CFU/mL of *Bacillus cereus* inoculum applied to *Festuca arundinacea*, while Z0, Z1, Z2, Z3, and Z4 represent the concentrations of 0, 10^3^, 10^5^, 10^7^, and 10^9^ CFU/mL of *Bacillus cereus* inoculum applied to *Poa pratensis*. Different letters represent notable differences between the treatments at *p* < 0.05. The bars are standard deviation in the table.

**Table 2:** Alpha diversity index of soil bacterial community.

Treatment			Chao1 Index	Shannon Index	Simpson Index	Coverage
*Festuca arundinacea* ‘Arid Ⅲ’	Non-rhizosphere soil	G0	649.76 ± 2.23 ^i^	7.27 ± 0.03 ^g^	0.956 ± 0.005 ^f^	0.987
		G1	687.29 ± 2.38 ^h^	7.40 ± 0.02 ^f^	0.977 ± 0.005 ^ab^	0.990
		G2	845.55 ± 4.93 ^d^	7.61 ± 0.04 ^d^	0.980 ± 0.001 ^a^	0.998
		G3	729.80 ± 5.27 ^g^	7.45 ± 0.01 ^e^	0.971 ± 0.002 ^c^	0.986
		G4	752.73 ± 3.89 ^f^	7.59 ± 0.06 ^de^	0.975 ± 0.003 ^b^	0.993
	Rhizosphere soil	G0	1025.64 ± 2.76 ^a^	8.57 ± 0.03 ^a^	0.966 ± 0.001 ^d^	0.988
		G1	1003.04 ± 3.86 ^b^	8.56 ± 0.04 ^a^	0.960 ± 0.001 ^e^	0.997
		G2	820.59 ± 3.58 ^de^	8.48 ± 0.04 ^b^	0.961 ± 0.002 ^e^	0.998
		G3	795.01 ± 3.72 ^e^	7.95 ± 0.03 ^c^	0.958 ± 0.003 ^ef^	0.988
		G4	973.20 ± 4.06 ^c^	8.52 ± 0.02 ^ab^	0.965 ± 0.002 ^d^	0.989
*Poa pratensis* ‘Midnight Ⅱ’	Non-rhizosphere soil	Z0	601.36 ± 2.58 ^h^	6.67 ± 0.04 ^h^	0.957 ± 0.003 ^g^	0.990
		Z1	646.55 ± 3.40 ^f^	6.75 ± 0.02 ^g^	0.962 ± 0.002 ^f^	0.998
		Z2	761.03 ± 4.23 ^e^	6.94 ± 0.01 ^ef^	0.988 ± 0.001 ^a^	0.988
		Z3	816.12 ± 1.917 ^d^	7.33 ± 0.02 ^c^	0.965 ± 0.001 ^e^	0.993
		Z4	873.15 ± 2.99 ^b^	7.91 ± 0.03 ^a^	0.975 ± 0.001 ^d^	0.994
	Rhizosphere soil	Z0	916.03 ± 7.91 ^a^	7.75 ± 0.02 ^b^	0.980 ± 0.003 ^b^	0.986
		Z1	828.77 ± 4.80 ^c^	7.72 ± 0.03 ^b^	0.981 ± 0.005 ^b^	0.989
		Z2	643.00 ± 2.74 ^f^	7.03 ± 0.02 ^e^	0.979 ± 0.002 ^bc^	0.985
		Z3	910.36 ± 4.69 ^ab^	6.89 ± 0.02 ^f^	0.975 ± 0.002 ^d^	0.991
		Z4	634.33 ± 2.71 ^g^	7.14 ± 0.04 ^d^	0.977 ± 0.002 ^c^	0.992

Note: G0, G1, G2, G3, and G4 represent the concentrations of 0, 10^3^, 10^5^, 10^7^, and 10^9^ CFU/mL of *Bacillus cereus* inoculum applied to *Festuca arundinacea*, while Z0, Z1, Z2, Z3, and Z4 represent the concentrations of 0, 10^3^, 10^5^, 10^7^, and 10^9^ CFU/mL of *Bacillus cereus* inoculum applied to *Poa pratensis*. Different letters represent notable differences between the treatments at *p* < 0.05. The bars are standard deviation in the table.

**Table 3:** The physicochemical properties of the tested soil.

pH	Organic Matter (OM, g/kg)	Total Nitrogen (TN, g/kg)	Total Potassium (TP, g/kg)	Total Carbon (TC, g/kg)	Available Phosphorus (AP, mg/kg)	Total Cd (T-Cd, mg/kg)
4.32	9.26	0.40	25.06	6.17	4.58	2.31

**Table 4:** Design of pot experiments.

Lawn Plants	Experimental Code	Inoculation Dosage	Viable Count
*Poa pratensis* ‘Midnight Ⅱ’	Z0	20 mL	0 CFU/mL
	Z1	20 mL	10^3^ CFU/mL
	Z2	20 mL	10^5^ CFU/mL
	Z3	20 mL	10^7^ CFU/mL
	Z4	20 mL	10^9^ CFU/mL
*Festuca arundinacea* ‘Arid Ⅲ’	G0	20 mL	0 CFU/mL
	G1	20 mL	10^3^ CFU/mL
	G2	20 mL	10^5^ CFU/mL
	G3	20 mL	10^7^ CFU/mL
	G4	20 mL	10^9^ CFU/mL

Note: G0, G1, G2, G3, and G4 represent the concentrations of 0, 10^3^, 10^5^, 10^7^, and 10^9^ CFU/mL of *Bacillus cereus* inoculum applied to *Festuca arundinacea*, while Z0, Z1, Z2, Z3, and Z4 represent the concentrations of 0, 10^3^, 10^5^, 10^7^, and 10^9^ CFU/mL of *Bacillus cereus* inoculum applied to *Poa pratensis*.

## Data Availability

Data are contained within the article.
